# Age-Associated Changes in the Immune System and Blood–Brain Barrier Functions

**DOI:** 10.3390/ijms20071632

**Published:** 2019-04-02

**Authors:** Michelle A. Erickson, William A. Banks

**Affiliations:** 1 VA Puget Sound Healthcare System, Geriatric Research Education and Clinical Center, Seattle, WA 98108, USA; 2 Division of Gerontology and Geriatric Medicine, Department of Medicine, University of Washington, Seattle, WA 98104, USA

**Keywords:** blood–brain barrier, aging, inflammation

## Abstract

Age is associated with altered immune functions that may affect the brain. Brain barriers, including the blood–brain barrier (BBB) and blood–CSF barrier (BCSFB), are important interfaces for neuroimmune communication, and are affected by aging. In this review, we explore novel mechanisms by which the aging immune system alters central nervous system functions and neuroimmune responses, with a focus on brain barriers. Specific emphasis will be on recent works that have identified novel mechanisms by which BBB/BCSFB functions change with age, interactions of the BBB with age-associated immune factors, and contributions of the BBB to age-associated neurological disorders. Understanding how age alters BBB functions and responses to pathological insults could provide important insight on the role of the BBB in the progression of cognitive decline and neurodegenerative disease.

## 1. Introduction

Advances in modern medicine, nutrition, hygiene, and safety standards have doubled the life expectancy of humans worldwide over the last century and a half [1]. It has been estimated that in the next 50 years, the elderly will comprise approximately 20% of the world population [2]. Therefore, it is imperative that the scientific and medical communities investigate approaches that will minimize age-associated disease and maximize quality of life. Age-associated neurological and neurodegenerative diseases are especially debilitating to the afflicted and their families, having tremendous emotional and socioeconomic costs. Changes in the immune system have long been recognized to occur with aging, and it is now appreciated that neuroinflammation likely contributes to age-associated neurological diseases [3]. However, it is less well understood how specific changes in the immune system with aging may affect central nervous system (CNS) functions and contribute to neurological disease. We posit that brain barriers, especially the blood–brain barrier (BBB) and blood–CSF barrier (BCSFB), are important interfaces between CNS and peripheral tissues that are affected by age-associated changes in the immune system. The BBB/BCSFB may, in turn, affect homeostatic functions of the CNS, and/or exhibit more detrimental responses to pathological stimuli. In this review, we will provide a brief overview of changes known to occur in the peripheral immune system with aging, and then discuss recent works that have explored the relationships of BBB/BCSFB dysfunction, healthy aging, and the immune system. We will also briefly discuss how age might contribute to BBB/BCSFB dysfunction in different disease states.

## 2. Changes in the Immune System with Aging

Aging is associated with immune-related changes that come with clinical consequences. For example, as one ages, vulnerability to certain infections increases, and effectiveness of many vaccines decreases [4]. These clinical features of aging are attributed to an overall decline in protective immune responses, termed “immunosenescence” [1]. Aging is also associated with low-grade inflammation that occurs in the absence of overt infection, termed “inflammaging” [5]. Immunosenescence and inflammaging are interrelated processes [6], and may occur with age due to a number of factors that include latent infections, metabolic changes, and cell/tissue injury. Changes in the adaptive and innate immune systems, and related physiological processes that are detectable outside of the CNS are summarized below. Later sections will further discuss known relations of these changes to neuroimmune functions of the BBB and BCSFB.

### 2.1. Age-Associated Changes in the Adaptive Immune System

The main function of the adaptive immune system is to confer immunological memory to the organism, which facilitates the rapid recognition and neutralization of specific pathogens upon subsequent encounters. Changes in the cellular arm of the adaptive immune system with age have been described comprehensively by many groups [7,8,9]. One prominent feature of immunosenescence in the elderly is the change in T-cell composition. In particular, there is a decrease in the number of naïve T lymphocytes and an increase in memory and effector T cells with age, as well as a reduced diversity in T-cell receptors, and diminished functions of both naïve and memory T-cells [7,10]. The mechanistic underpinnings of these changes have been described elsewhere [7]. Changes in the B-cell compartment include reduced B-cell numbers, a reduced repertoire of B-cell receptors, reduced proliferative capacity, and decreases in immunoglobulin class-switch recombination [10,11]. The immunological consequences of reduced B-cell and T-cell functions include the reduced ability to generate immune memory to novel antigens, and thus, the reduced vaccine efficacy and increased vulnerability to certain infections in the elderly. 

### 2.2. Age-Associated Changes in the Innate Immune System

The innate immune system is important for mounting initial protective responses against infections, and in sterile tissue injury and wound repair. The innate immune system also initiates cross-talk with the adaptive immune system through antigen presentation, co-stimulatory molecule expression, and cytokine production and so can contribute to adaptive immune responses [12]. The major changes in the innate immune system with aging include a heightened level of baseline inflammation, and an impaired ability to mount an efficient innate immune response against pathogenic stimuli. Specific changes in the function of innate immune cells have been comprehensively described elsewhere [13], and include impairments in phagocytosis, capacity to produce reactive oxygen and nitrogen species, T-cell priming, and signaling through pattern recognition receptors. 

### 2.3. Age-Associated Changes in the Microbiome

A significant portion of the body’s immune system resides in or near the gastrointestinal tract and can regulate the resident gut microbial populations. In young humans, the most numerous and diverse bacterial phylum is Firmicutes, with most in this phylum belonging to the Clostridia class. The second most abundant phylum is Bacteriodetes, which shows a high level of subject-to-subject variability in phylotypes detected [14]. In initial studies from the ELDERMET consortium that explored differences in gut microbial populations in young versus elderly subjects, it was found that elderly subjects had a lower proportion of Firmicutes, and atypical Bacteriodetes/Firmicutes ratios where Bacteriodetes predominated [15]. However, this study and others also demonstrated that elderly subjects show high variability in their microbiota profiles [15,16]. Notably, many of the Firmicutes are major producers of the short chain fatty acid, butyrate [17], which has histone deacetylase inhibitor activities and has been shown to protect against age-associated conditions such as sarcopenia and cognitive impairment in rodent models of neurodegenerative disease [18,19]. Increases in pathogenic bacteria that thrive in pro-inflammatory environments such as streptococci, staphylococci, enterococci, and enterobacteria have also been reported with aging [16], namely in centenarians [20]. 

Gut-associated lymphoid tissues (GALT) are the major immune interfaces of the gut that regulate the microbiome throughout lifespan. Age-associated changes in GALT include a reduction in antigen-specific IgA-immune responses which are, in part, mediated by aberrant cytokine responses of CD4+ T-cells [21]. It has also been shown that aging is associated with a reduced induction of immune tolerance to novel oral antigens [22]. Loss of the immunoregulatory environment within the gut with aging may have consequences, such as immune responses to novel antigens that would normally be tolerated, or a shift in gut microbial populations [16]. A pro-inflammatory environment within the gut could also result in microbial translocation and release of pathogenic microbes and/or their products (e.g., lipopolysaccharides) into the bloodstream, which could affect distal organs such as the CNS [23,24].

Dietary and environmental changes that are specifically associated with aging may also contribute to alterations in the microbiome. For example, dietary changes may occur upon new residence in assisted living institutions [16]. The elderly are also disproportionately affected by *Clostridium difficile* infection, and risk factors that may facilitate changes in the microbiome include increased use of antibiotics, and prior health care exposures where *C. difficile* may be contracted [25].

### 2.4. Age-Associated Changes in Peripheral Tissue Microenvironments and the Circulation

The transition of cells to a senescent phenotype is thought to be a protective mechanism against malignancies, and accumulation of senescent cells in multiple tissues occurs with aging [26]. Senescent cells are growth-arrested, but they remain metabolically active and undergo dramatic changes in protein expression and secretion, primarily in response to DNA damage [27]. The senescence-associated secretory phenotype (SASP) involves secretion of soluble cytokines, chemokines, and growth factors, proteases, extracellular matrix components, and reactive oxygen and nitrogen species which together modify the tissue microenvironment to promote local inflammation and tissue damage [26]. Therefore, cellular senescence may be one contributing factor to inflammaging. It has been proposed that cells of the CNS that have proliferative capacities such as endothelial cells and glia may also adopt a SASP, which could result in low-grade inflammation in the aging brain [28].

The BBB could also be affected by the accumulation of SASP cells in the periphery if exposed to pro-inflammatory secreted factors in the bloodstream. Many studies to date have demonstrated elevations in circulating inflammatory and acute phase proteins with aging [29,30,31,32]. We have also recently reviewed many aspects of neuroimmune interactions of the BBB and BCSFB with immune factors associated with SASP [33]. Recently, a novel aptamer-based proteomic approach was used to assess proteomic profiles in blood with healthy aging [34]. This study significantly detected an overall enrichment of SASP proteins in blood with aging, although some classical aging biomarkers such as interleukin-6 (IL-6), tumor necrosis factor-α (TNF-α), and insulin-like growth factor-1 (IGF-1) were not among the top-ranking age-associated proteins [34,35]. However, the proteins detected did reflect enriched signaling pathways such as cytokine/cytokine-receptor interactions, complement and coagulation cascades, and axon guidance. Notably, the protein that most strongly correlated with aging in this study was macrophage inhibitory cytokine-1 (MIC-1)/growth differentiation factor 15 (GDF15), which is a transforming growth factor-β (TGF-β) superfamily member that has anti-inflammatory activities in vitro [34,36]. Recent studies have also implicated GDF15 in obesity and the regulation of body weight, as well as frailty [37,38,39]. Overall, results from biomarker studies suggest that there may be differences in blood biomarkers that could signify healthy aging and predict SASP-associated disease.

## 3. Age-Associated Changes in Neuroimmune Functions

### 3.1. Altered Neuroimmune Phenotypes with Aging

Evidence supports that aging causes a pro-inflammatory environment in the CNS. Factors that have been proposed to contribute to increased baseline activation of inflammatory processes in the brain include reactive oxygen species, release of damage-associated molecular patterns (DAMPs) from injured or dying cells, increased abundance of cells with SASP phenotypes, and responses to peripheral inflammatory signals [40,41,42,43,44]. In humans, non-human primates, and rodents, aging is associated with increased numbers of reactive microglia and astrocytes [45]. The reactive phenotype of both astrocytes and microglia is typically determined by the expression levels of specific cell surface markers, as well as morphological changes of the cells. For example, there is an increased proportion of microglia in aged mice that stain positive for cell surface markers such as major histocompatibility complex II (MHCII), and cluster of differentiation (CD)11b, 86, and 68 [46]. Microglia in the healthy brain adopt a ramified morphology, characterized by long, branched extensions from the cell body that function in surveying the local environment [47]. With aging, microglia de-ramification is apparent: processes retract and thicken, and cell bodies enlarge [46,48]. Astrocytes also demonstrate morphological changes and increased expression of the inflammatory surface marker, glial fibrillary acidic protein (GFAP), with aging [46]. Aging also involves a shift in cytokine expression profiles, with increases in pro-inflammatory cytokines such as interleukin-1β (IL-1β) and IL-6, and decreases in anti-inflammatory cytokines such as interleukins 10 and 4 (IL-10 and IL-4) [46]. Overall, these pro-inflammatory phenotypes of glia at baseline are thought not only to reflect chronic, low-grade neuroinflammation, but also a “primed” phenotype whereby glia have more robust responses to immune stimuli [44]. 

### 3.2. Altered Neuroimmune Responses to Stimuli with Aging

It is appreciated that the aging brain may be more vulnerable to pathological changes in response to acute illness and infections. For example, urinary tract infections, which are not associated with cognitive symptoms in the young, can cause delirium and other neuropsychiatric conditions in the elderly [49]. Rodent models also suggest that aged mice have more severe neuroinflammatory responses and exacerbated behavioral outcomes following peripheral immune stimuli [50]. A prototypical stimulator of the innate immune system is lipopolysaccharide (LPS), which is a cell wall constituent of Gram-negative bacteria that activates inflammatory signaling cascades through the pattern recognition receptor Toll-like receptor 4 (TLR4) [51]. Young, healthy mice treated intraperitoneally with LPS exhibit a systemic cascade of cytokines and chemokines in the blood and brain [52], reactive gliosis, changes in body temperature and weight, and sickness behaviors. Intraperitoneal injection of LPS in aged mice causes increased pro-inflammatory cytokine responses and reactive microgliosis versus young mice [50,53,54]. Behavioral complications of peripheral infections and/or exposure to bacterial components is also more pronounced with age. For example, aged rodents are more vulnerable to cognitive impairment, sickness behavior, and depressive-like behavior following exposure to systemic inflammatory stimuli [50,54,55,56]. Neuroinflammatory stimuli, such as injection of the cytokines TNF-α and interferon-γ (IFN-γ) in the lateral ventricle, also result in increased reactive gliosis which occurs in the absence of apparent neurodegenerative changes [57].

## 4. The BBB as an Interface for Neuroimmune Communication

### 4.1. Anatomical, Cellular, and Subcellular Organization of Brain Barriers

#### 4.1.1. The Vascular BBB

The primary anatomical unit of the vascular BBB is the brain endothelial cell (BEC). BECs have unique phenotypic properties that restrict the unregulated diffusion of molecules from blood into brain (barrier functions), and also those that regulate the passage of circulating nutrients, hormones, peptides, and proteins into and out of the brain (transport functions). In addition, the vascular BBB is an important signaling and secretory interface and uniquely regulates immune surveillance in the brain [33]. In Section 4.2, we will discuss how these functions of the BBB contribute to the immune-privileged status of the CNS and to unique aspects of neuroimmune communication.

Barrier functions of the vascular BBB are conferred by at least four distinct phenotypes of brain endothelial cells. These include expression of specialized tight junction proteins, reduced levels of pinocytosis, expression of efflux transporters, and expression of metabolic enzymes Tight junction protein complexes expressed by BECs localize to cell–cell junctions and prevent the diffusion of substances between cells (paracellular diffusion). Tight junctions are comprised of integral membrane proteins that include claudins (namely, claudin-5), occludin, and junctional adhesion molecules such as zonula occludens [58,59]. In addition to limiting paracellular diffusion, tight junctions can limit the lateral diffusion of membrane proteins and thus confer polarity to BECs. Tight junction proteins also interact with the cytoskeleton, adherens junctions, and the extracellular matrix, and are dynamically regulated by a range of stimuli at transcriptional and post-translational levels [58]. Relative reductions in fluid-phase pinocytosis also contribute to the BBB, and recent works have begun to elucidate molecular processes that are uniquely active in BECs and suppress formation of pinocytic vesicles. For example, the lipid transporter major facilitator superfamily domain containing 2A (Mfsd2a) confers a unique membrane lipid composition to brain endothelial cells that prevents assembly of caveolin-1 vesicles [60,61]. Finally, the BBB expresses specialized efflux transporters and metabolic enzymes that prevent the diffusion of circulating xenobiotics and other molecular substrates that would otherwise accumulate in the brain. Most efflux transporters at the BBB belong to the family of ATP-binding cassette transporters, and include P-glycoprotein (P-gp), multidrug resistance proteins (MRPs) and breast cancer resistance proteins (BCRPs) [62]. Examples of metabolic enzymes that contribute to BEC barrier functions include those that metabolize neurotransmitters (e.g., monoamine oxidases, cholinesterases, and aminopeptidases), and Phase I and II enzymes such as cytochrome P450s and transferases that are important for drug metabolism [63].

Like peripheral organs, the brain derives nutritive and trophic support from the circulation. However, energy and anabolic substrates such as glucose and amino acids that are derived from the circulation do not freely diffuse across BEC membranes, and so require transporters at the BBB to permit their passage from brain-to-blood in sufficient concentrations to support normal brain functions. Similarly, peptides and proteins such as insulin, leptin, ghrelin and some cytokines and chemokines can cross the intact BBB, and utilize specialized transport systems to do so. Transport systems at the BBB include solute carriers, which facilitate energy-independent transport down a concentration gradient, endocytic receptors, which bind ligands and transport them from one side of the membrane to the other in an energy-dependent process, and adsorptive endocytosis which involves interactions with the glycocalyx [33]. BBB transporters are important for conveying signals that relay aspects of metabolic status such as satiety and adipose mass, as well as inflammatory status which will be discussed in greater detail in Section 4.2. Also described in greater detail in Section 4.2 are the signaling and secretory interface functions of the BBB and their relevance to neuroimmune communication.

The specialized phenotype of BECs is greatly influenced by their local environment and closely associated supportive cells that are collectively termed the neurovascular unit (NVU). The most closely apposed cells to BECs are pericytes, which are found mostly around capillaries and post-capillary venules, and share a basement membrane with the brain endothelium. Pericytes are important for BBB induction and maintenance, as has been shown in mouse models with pericyte deficiencies [64]. Astrocyte end feet are also in very close proximity to the BBB, and ensheath the vessels. Astrocytes are also important for BBB induction and maintenance, as astrocyte conditioned medium is sufficient to promote BBB properties of BECs cultured in vitro [65]. Other components of the NVU include neurons, microglia, oligodendrocytes, and the extracellular matrix, which have been described for their contributions to BBB function under physiological and inflammatory states [33]. 

#### 4.1.2. The Epithelial BCSFB

The BCSFB exists at the level of brain epithelial cells that comprise the choroid plexus (CP), which is located in each of the brain ventricles. Arachnoid epithelial cells also contribute to the BCSFB. Notably, endothelial cells comprising the vasculature in the CP do not have a BBB phenotype, and so permit leakage of serum components into the CP stroma [66]. The CP vasculature is also permissive to leukocyte trafficking, and so the stroma within the CP is a site where immune surveillance actively occurs. The CP epithelial cells of the BSCFB, similar to the BBB, express specialized tight junction proteins and efflux transporters that contribute to the barrier properties of the choroid plexus epithelium (CPE). The tight junction protein repertoire of the CPE is somewhat distinct from BECs in that they are comprised of distinct claudin proteins (1, 2, and 11) [59]. The CPE is the major site of cerebrospinal fluid (CSF) production, and CPE transporters are important for regulating CSF composition (reviewed in [67]). The arachnoid epithelium, while not a site for CSF production, does express the tight junction protein claudin 11, and efflux transporters such as P-gp and BCRP which may influence drug penetration in to the brain [33].

In contrast to the brain parenchyma, which has very low levels of blood-derived leukocytes under physiological conditions, the CSF and meninges do have resident populations of blood-derived leukocytes which must cross brain barriers to enter these compartments [68]. Indeed, the choroid plexus and arachnoid epithelial cells of the BCSFB are proposed to be major routes by which leukocytes gain entry to CSF under healthy conditions [68,69], and can also be a route of entry in injured states [70]. Aspects of leukocyte trafficking to the brain across brain barriers with aging will be discussed later in Section 5.1.4. 

### 4.2. Neuroimmune Axes of the BBB

The BBB prevents the unregulated exchange of neuroimmune substances and cells between the CNS and blood. Hence, it is the BBB more than any other structure that secures the CNS as an immune-privileged tissue. However, the immune-privileged status of the CNS is relative as a number of mechanisms establish links between the peripheral components of the immune system and those of the CNS. These mechanisms are operational physiologically and, as discussed below, can be involved in aging and in aging-related diseases. Some of these mechanisms, such as vagal and other cranial nerve afferents, do not directly involve the BBB, whereas many others do. For convenience, mechanisms of BBB-neuroimmune interactions can be grouped into five categories or “axes” [33].

The first axis relates to the physiological regulation of the barrier properties that prevent leakage and is currently the least understood of the axes. As discussed above, much is known about how the barrier [71] is formed and even about how it can breakdown to once again become leaky [72,73,74,75,76]. However, there is some evidence that a degree of leakage may occur normally, if transiently. Hormones known to affect BBB tightness and that vary diurnally or with aging include insulin and dehydroepiandrosterone [77,78]. Such “physiological” leakage is probably at a very low level and its purpose is unknown. 

A second axis is the alteration of other barrier functions, such as its transporter functions, by neuroimmune substances. There are many examples of these, such as TNF-α affecting the brain endothelial cell cytoskeleton [79], LPS increasing insulin transport [80], and granulocyte-macrophage colony-stimulating factor and IL-6 modulating BBB permeability to human immunodeficiency virus [81].

A third axis relates to the ability of the barriers to transport neuroimmune substances between the CNS and the blood. The best studied in this category are blood-to-brain transporters for cytokines, including IL-1α and β, IL-6, and TNF-α [82]. 

A fourth axis relates to immune cell trafficking as discussed above. This axis is clearly involved in both disease, as exemplified by multiple sclerosis, and in normal brain functioning [83]. The latter is illustrated by the belief that an impairment of immune cell surveillance in the brain can lead to progressive multifocal leukoencephalopathy [84].

The fifth axis relates to the ability of barrier cells themselves to secrete neuroimmune substances. For example, LPS acting at the luminal surface of brain endothelial cells induces release of prostaglandins into brain [85,86], resulting in fever. Barrier cells also secrete nitric oxide and cytokines [87]. Such release can be constitutive or induced. Secretion can be either from the same cell membrane surface (i.e., luminal–luminal or abluminal–abluminal) that receives the immune stimuli or, as in the case of LPS-prostaglandin-fever, from the opposite cell membrane surface [88].

These axes can interact in dynamic ways. As discussed below, they are known in some cases to be involved in aging and aging-related diseases.

## 5. Neuroimmune Mechanisms of Age-Associated Changes at the BBB

As was conveyed in Section 2, physiological aging is associated with changes in the immune system that may occur in response to the altered molecular environment of the aged organism. Although very few studies to date have explored direct relationships between BBB dysfunction and age-associated changes in peripheral components of the innate or adaptive immune systems (discussed in Section 5.1.5), emerging works have explored mechanistic changes at the BBB with aging that may contribute to altered neuroimmune functions. In this section, we will discuss changes at the BBB that are associated with aging in the absence of overt disease, and how physiological aging may affect BBB responses to immune stimuli. We will also consider activities of age-associated signaling pathways at the BBB and BCSFB, and how these might be affected using pharmacological approaches. 

### 5.1. Changes in Brain Barrier Function with Aging

A challenge in the assessment of BBB dysfunction in healthy human aging is that many parameters can only be assessed in post-mortem tissues, and so it is difficult to distinguish changes at the BBB in humans that occur as a result of aging versus disease. Measurements of BBB dysfunction in living human subjects using imaging techniques such as PET, SPECT, and MRI are also becoming more robust with advances in instrumentation and analysis techniques, and have suggested that pathological changes at the BBB do occur progressively with aging, and predict clinical symptoms such as cognitive impairment. Findings in rodent models also corroborate general aging-associated phenotypes of the BBB and have elucidated possible mechanisms by which BBB functions are altered with age. These details are further described below. 

#### 5.1.1. Brain Barrier Disruption

One of the most-studied (and yet, poorly understood) aspects of BBB dysfunction is disruption [89], which is typically defined by the apparent leakage of normally BBB impenetrant molecules. Recent imaging results argue that BBB disruption does occur in healthy aging, and is worse in individuals with mild cognitive impairment, which is considered a prodrome of Alzheimer’s disease (AD) [90,91]. One common approach to proxy BBB disruption in living humans is to measure the ratio of abundant, BBB-impermeant proteins such as albumin or immunoglobulin G (IgG) in CSF versus serum. However, these measures may be confounded by other known CNS deficits with aging, such as altered production and reabsorption of CSF, and inflammatory changes in the serum and CSF levels of these proteins, which have been discussed previously [57,92]. Further, there may be leakage of the BCSFB and altered protein synthesis at this site with age [93,94]. Recent studies have implemented advanced imaging technologies that can visualize leakage of intravenously injected tracers such as gadolinium via dynamic contrast MRI, and these have indicated that vascular BBB disruption does occur in the aging human brain, albeit at low levels [91]. 

In healthy aged mice (24 mo.), leakage of IgG into the parenchymal space of the cerebral cortex and hippocampus occurs when compared with young mice (3 mo.), suggesting that there is BBB disruption in this model. Increased IgG leakage in aged mice was associated with astrogliosis, endoplasmic reticulum (ER) stress, and increased endothelial cell levels of TNF-α; the latter measure significantly correlated with circulating levels of IL-6. In the same study, a significant reduction in occludin expression per brain endothelial cell was also observed in aged mice [95]. Other studies have corroborated findings of BBB disruption in aging mice [96]. Molecular mechanisms of BBB disruption in aging have been identified, and include reduced expression of sirtuin-1 [96], a de-acetylase enzyme which has been implicated in the regulation of lifespan, senescence, and inflammatory responses to environmental stress [97]. 

BBB disruption in the context of aging or disease could result in disease exacerbation through leakage of potentially harmful proteins into the brain [91]. However, it is not entirely clear that BBB disruption under any circumstance will always lead to brain damage. For example, certain therapeutic strategies for delivery of chemotherapeutics to the brain have relied on transiently disrupting the BBB, and are generally well-tolerated when brain cancers are the target [98]. Recent work has also indicated that repeated transient BBB disruption in humans with AD using focused ultrasound did not cause any serious clinical or radiological adverse events [99]. In contrast, healthy rodents with no prior brain abnormalities showed symptoms of reactive gliosis and neurodegeneration when transiently perfused with mannitol to cause widespread disruption of the BBB [100], and also had increased deposition of harmful serum proteins like fibrinogen in the CNS [101,102]. The apparent paradox in efforts to disrupt the BBB as a therapeutic strategy versus BBB disruption having known adverse consequences on the CNS and associations with many CNS diseases highlights the complexities of BEC barrier functions that are likely nuanced and context-specific. Why BBB disruption in and of itself is apparently innocuous under some conditions, but clearly detrimental in others remains to be understood in greater molecular detail.

#### 5.1.2. Transporter Dysfunctions and Altered Signaling at Brain Barriers with Aging

Glucose transport: Glucose is the main energy source for the brain. The BBB regulates glucose uptake by the brain through expression of the glucose transporter GLUT1 on brain endothelial cells. GLUT1 is a uniporter that facilitates glucose diffusion from blood-to-brain. The amount of glucose uptake into brain is thus thought to depend on energy utilization by neurons which maintains a concentration gradient that drives glucose diffusion into the brain [103]. Under this assumption, neuronal dysfunction or neurodegeneration would result in reductions of glucose uptake by the brain due to reduced energy utilization and thus loss of the glucose concentration gradient. However, more recent works have suggested that reductions in brain glucose uptake could also reflect BBB dysfunction in glucose transport [103,104]. Brain glucose uptake can be measured in humans by imaging the uptake of ^18^F-fludeoxyglucose into the brain with PET. Using this technique, it was shown that there is reduced glucose uptake into the brain in the frontal and temporal cortex with aging, even after correction for volume loss [105]. Aged rodents also show reduced brain glucose uptake, which is associated with cognitive impairment [106,107]. In mice, GLUT1 reductions at the BBB are apparent at 15 mo., and are even further reduced in an AD model of the same age [108].

Amyloid beta transport: Accumulation and deposition of the amyloid beta (Aβ) protein in the brain is a pathological hallmark of AD and contributes to neurodegeneration [109]. The BBB expresses transport systems for Aβ that mediate both transport into (influx) and transport out of (efflux) the CNS. Efflux transporters are thought to be important regulators of Aβ clearance from the brain, and these include the low-density lipoprotein receptor-related protein 1 (LRP-1) and P-gp [110,111,112]. The latter is also an important multidrug efflux transporter that can affect drug delivery to the brain. LRP-1 expression was shown to be decreased in brain microvessels with age, and in AD [110,113]. P-gp function is decreased in aged humans [114,115,116] as well as in aged mice [117]. Collectively, these changes at the BBB with age could contribute to Aβ accumulation in the brain with AD. It is also known that systemic inflammation in young mice can contribute to Aβ efflux deficits [118]; whether there is an inflammatory component to the Aβ efflux deficit in aging remains to be determined. 

Insulin transport: Insulin is a trophic factor in the brain, and regulates critical functions such as feeding and learning and memory [119]. Brain insulin is not thought to be derived from CNS production, but rather from circulating insulin produced by the pancreas. Transport of insulin from blood-to-brain occurs through saturable transport mechanisms at the BBB [120,121,122]. In humans, it was recently shown that CSF/serum ratios of insulin decrease with aging [123], suggesting that BBB transport may be impaired. Reductions of insulin concentrations in brain tissues have also been reported with human aging [124]. In the senescence-accelerated mouse P8 (SAMP8) model of accelerated aging and AD-like cognitive decline, significant differences in the transport rate of insulin across the BBB were not observed in young versus aged mice. Increased insulin occupancy of vascular space was observed in aged SAMP8 mice in the parietal cortex, cerebellum, and thalamus, which indicates that there may be increased binding of insulin to brain endothelium with age in these regions [125]. It has not yet been determined whether commonly used mouse strains exhibit alterations in insulin transport across the BBB with age.

#### 5.1.3. Interactions of Age-Associated Circulating Factors with Brain Barriers

Aging is associated with both increases in circulating factors that are harmful to the CNS, and decreases in circulating factors that are protective [29,126]. For example, circulating levels of growth differentiation factor 11 (GDF11, a member of the TGF-β superfamily), decline with age [127], and GDF11 treatments can stimulate vascular proliferation in vitro and in the subventricular zone of aged mice [128]. A circulating factor that increases in blood with aging is the chemokine CCL11, which has been shown through parabiosis studies to mediate cognitive impairment and to inhibit neurogenesis [29]. CCL11 in the circulation can access the CNS through a non-saturable or high capacity transport system at the BBB [129], indicating that increasing levels of circulating CCL11 in blood with age contribute to increased brain levels even when the BBB is intact. Another recent report has demonstrated that the enzyme acid sphingomyelinase (ASM) can contribute to BBB dysfunction [130]. This study showed that ASM concentrations increase in the circulation and in brain endothelial cells with aging. When compared with old mice that had reduced capillary density and evidence of BBB disruption, it was shown that mice heterozygous for sphingomyelin phosphodiesterase 1 (*Smpd1*) gene, which encodes ASM, were protected against these age-associated changes. It was further shown that ASM contributes to BBB disruption through induction of caveolae-cytoskeleton interactions that result in increased fluid-phase pinocytosis, but not through any apparent changes in paracellular/tight junction-regulated routes. ASM has enzymatic activity that facilitates the hydrolysis of sphingomyelin to ceramide and phosphorylcholine conversion [131], and thus altered membrane lipid composition could be contributing to the apparent changes in pinocytosis as well. Smpd1 heterozygosity also protected against age-associated deficits in learning and memory [130]. 

**Figure 1:**
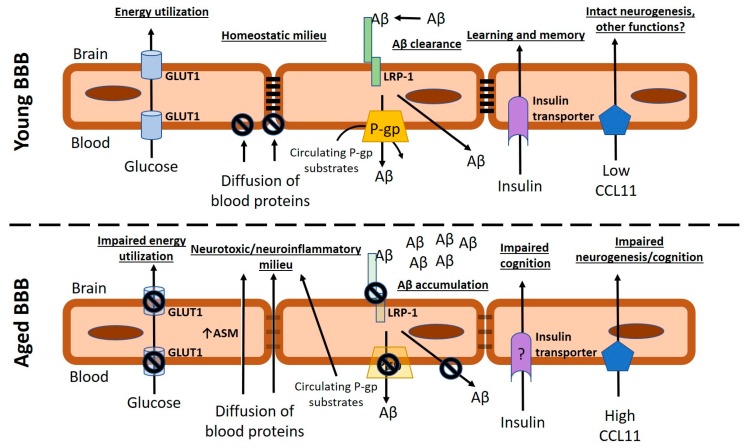
Changes in vascular blood–brain barrier (BBB) function that may lead to brain pathology with age. The upper panel depicts physiological functions of the BBB such as glucose transport, expression of intact tight junction complexes and suppression of vesicular processes that prevent the paracellular or transcellular leakage of blood proteins into the brain, intact functions of the efflux transporter P-gp, which contributes to barrier function by limiting diffusion of its substrates into the brain, and in concert with lipoprotein receptor-related protein 1 (LRP-1), facilitates amyloid beta (Aβ) clearance from the brain. Additionally, entry of insulin into the brain supports neuronal functions and contributes to learning and memory, and brain entry of circulating compounds with high-capacity transporters such as CCL11 is limited by low circulatory concentrations. The lower panel depicts aspects of BBB dysfunction that are either supported or suggested to occur with aging. Transparent appearance of transporters (GLUT1, LRP-1, and P-gp) indicates reduced protein expression levels at the BBB, and the interdictory circles over transporters indicate known functional impairments which may occur in the presence or absence of expression changes. The question mark on the insulin receptor suggests a possible mechanism by which aging influences brain insulin through altered function of the insulin transporter, which has not yet been definitively determined.

A summary of disruptive and non-disruptive changes at the vascular BBB with age and consequences to CNS function is depicted in Figure 1.

#### 5.1.4. Age-Associated Changes in Inflammatory Signaling at the Choroid Plexus 

The choroid plexus epithelium that comprises the BCSFB is an important immunological brain interface. The CSF is immunologically active, and contains cells of the adaptive immune system such as central memory T-cells which are thought to participate in CNS immune surveillance [132]. The BCSFB is an important site for leukocyte trafficking into CSF [132,133] and may regulate both protective and pathogenic types of immune cell recruitment to damaged tissues in the CNS and peripheral nervous system (PNS) [133]. Therefore, changes in BSCFB function with age may alter aspects of protective adaptive immunity in the CNS. We discuss this prospect in more detail in the following section. 

Age-associated changes in gene expression profiles of the CPE have been found in both mice and humans, and some of the most robust changes in CPE gene expression were related to interferon (IFN)- associated pathways. With aging, there is an increased expression of type I IFN-response genes, and a decrease in type II IFN-response genes at the CP [134]. In the same study, it was found that circulating factors from aged mice reduced type II IFN gene expression, whereas factors in CSF increased type I IFN gene expression, suggesting that the aging brain and systemic compartments have distinct effects on CP gene expression. In aged mice that demonstrated deficits in spatial memory, it was shown that blocking signaling of the interferon α/β receptor, which binds type I IFN cytokines, could improve spatial memory. Aging also induces a shift in cytokine levels expressed at the CP, with increased expression of IL-4 and pro-inflammatory cytokines IL-1β and IL-6, and reduced expression of the type II interferon IFN-γ. It was also found that CPEs could express CCL11, which is induced by IL-4, when IFN-γ levels are low [134]. Therefore, the CP in addition to blood could be a source of CNS CCL11. 

Another molecule thought to regulate the CPE with aging is the protein Klotho. Klotho is a transmembrane protein that facilitates signaling of fibroblast growth factor 23 (FGF23), and can also be secreted or cleaved from the membrane by a disintegrin and metalloproteinase domain-containing protein (ADAM) 10 or 17 and released as a soluble form to activate transient receptor potential cation channel subfamily V (TRPV5) signaling or inhibit IGF-1 and wnt signaling [134]. Mice lacking functional Klotho exhibit an accelerated aging phenotype which includes early thymic involution, osteopenia, skin atrophy, hearing loss, and neurodegeneration [135,136]. Klotho expression is not ubiquitous among tissues but is expressed at high levels in the CPE [136]. Expression of Klotho mRNA and protein is significantly reduced at the CPE with age [136]. Such reductions are also associated with increased expression of MHCII in CP stroma, increased levels of peripheral blood-derived macrophages in CP stroma and increased microglial activation, and NLRP3 inflammasome activation [137]. Therefore, Klotho may have important functions in suppressing activation of innate immunity in the CPE, and its reduction may be one mechanism by which neuroimmune functions change with age.

#### 5.1.5. Immune Cell Trafficking

Immune cell trafficking in the brain under healthy conditions is largely compartmentalized to CSF and meningeal spaces, and is thought to be mediated in part through expression of P-selectins and intracellular adhesion molecule-1 (ICAM-1) expressed by the choroid plexus and arachnoid epithelium [68,138]. The post-capillary venules of the BBB are also interfaces for immune cell trafficking, particularly in disease states such as brain injury and multiple sclerosis. Detailed aspects of immune cell trafficking across brain barriers have been discussed by us in a recent review [33]. Relatively little is known about how immune cell populations in the CNS change with healthy aging, or how brain barriers may regulate such changes. However, it is plausible that relations do exist, since changes in peripheral leukocyte populations occur with aging (discussed in Section 2), and brain barriers are active sites of immune cell trafficking to the CNS in both healthy and injured/diseased states. Further, it should be considered whether peripheral changes in innate and adaptive immune cell composition with age could have important implications for CNS function. T-cells, which are the major blood-derived leukocyte population in the CNS (mostly residing in the CSF and meninges) have recently been shown to regulate aspects of learning and memory [138], but it is presently unclear whether age-related changes in T-cell subsets are associated with cognitive deficits. 

Increases in T-cell and dendritic cell numbers have been observed in aged mice, starting at about 12 months [139]. One recent study in mice has explored relations among changes in leukocyte subsets in brain and blood [140]. The results of this study showed that numbers of T-cells, but not myeloid cells or other CD45+ cell types significantly increased in the brain with age. Further exploration of T-cell subsets demonstrated that in blood, the ratio of CD4+/CD8+ T-cells decreased with age, whereas age had no effect on the CD4+/CD8+ ratios in brain. In brain, the majority of T-cells detected were CD8+, and localized to perivascular spaces, brain parenchyma, and in the choroid plexus and meninges. Interestingly, the majority of T-cells in human CSF are CD4+ central memory T-cells [69], and so may reflect a different population than those found in brain parenchyma, although species differences may also explain the different abundances of CD4+ versus CD8+ T-cell subsets. The majority of the CD8+ T-cells in aged mouse brains had an effector memory phenotype, and the enrichment of these cells in the brain with age was not attributed to clonal expansion following exposure to brain antigens [140]. Age-associated increases in CD8+ T-cells positively correlated with microglia numbers, and phagocytosis, but negatively correlated with TNF-α positive microglia, suggesting that the CD8+ T-cells may be facilitating microglia polarization towards a phagocytic phenotype. However, it is also possible that the aged microglial phenotype could be driving T-cell recruitment. Finally, this study showed that CD8+ brain T-cells from aged mice produce greater levels of reactive oxygen species and pro-inflammatory cytokines following ischemic injury [140]. 

Overall, these data indicate that T-cell subsets in brain are distinct from those that predominate in blood, and that changes in T-cell subsets in aged blood are not necessarily reflected by the same population changes in the brain. Although it is presently not known which T-cell subsets in the circulation contribute to brain T-cell populations in parenchyma or CSF/meninges, findings from Ritzel et al. did indicate that T-cells from aged mice had elevated expression of adhesion molecules that are necessary for capture and diapedesis across brain barriers [140]. Future studies are needed to determine the contributions of brain barriers to age-associated increases in T-cell trafficking to the brain.

### 5.2. Effects of Aging on BBB Responses to Immune Stimuli

Dysfunction of the BBB can occur in concert with systemic and neuroinflammatory changes, however existing data suggest that the young, healthy BBB is relatively resistant to dysfunction caused by peripheral inflammatory insults, and relatively high doses of immune stimulators like LPS are required to elicit BBB disruption and dysfunction of transporters [118,141,142]. This is also supported in humans, where it was recently shown that in the absence of CSF abnormalities that would suggest disease, there were no correlations of systemic inflammatory markers with CSF/serum albumin ratios [143]. These findings further suggest that the healthy BBB of young adults is resistant to disruption induced by moderate systemic inflammation. 

It is also understood that BBB dysfunction in response to neuroinflammatory stimuli may be regulated by the systemic inflammatory context. For example, intracerebroventricular (ICV) injection of IL-1β causes a robust influx of leukocytes into CNS parenchyma, but an intraperitoneal dose of LPS inhibits the ability of ICV IL-1β to recruit leukocytes to the brain [144]. The apparent resistance of the BBB to leukocyte trafficking in the presence of systemic inflammation in this context could be an adaptive advantage to protect the CNS from the systemic response to pathogen infections in the periphery.

As previously discussed, aging is associated with decreased BBB integrity and functional impairment of transporters. Aging may also exacerbate BBB responses to CNS injury and systemic inflammatory stimuli. In an LPS model that causes cerebral microhemorrhages (CMH), it was shown that mice aged 18 months had more numerous and severe CMH than young mice. This phenotype was associated with increased microgliosis and astrogliosis [145]. Aging also can cause dramatic changes in sleep, which is associated with increased production of pro-inflammatory cytokines such as TNF-α [146]. Cytokines such as TNF-α can cross the intact BBB, and so peripherally derived TNF-α may enter the brain to activate neuroinflammatory responses directly [147]. Sleep fragmentation in aged mice significantly increased the transport of TNF-α into the brain, but had no significant effect in young mice [146]. 

### 5.3. Interactions of the BBB with the Aging Microbiome

Interactions of the microbiome with the BBB have been reviewed recently [148]. Much of the current knowledge of these interactions is based on findings in germ-free mouse models, which exhibit increased BBB disruption that is apparently due to reduced tight junction protein expression and tight junction dysfunction [149]. It is therefore plausible that changes in the microbiome with age may affect BBB function. To date, no studies have directly tested this possibility, although emerging works suggest potential mechanisms. For example, short-chain fatty acids such as butyrate have been shown to protect against BBB disruption in germ-free mice [149]. Emerging evidence also supports that there is a reduced capacity of the microbiome to produce butyrate in the elderly [150], and so it may be that reduced butyrate levels contribute to age-associated BBB dysfunction. Recent studies have also begun to identify how age-associated changes in the microbiome might affect aspects of CNS function in which the BBB could be involved. For example, aged mice have altered cecal microbiota compositions, which is associated with increased gut permeability and higher levels of circulating pro-inflammatory cytokines in the periphery versus young mice. The same aged mouse cohort also demonstrated increased anxiety-like behaviors and impaired object–place recognition memory and social recognition [151]. Although altered BBB functions were not examined in this study, future works could further elucidate relationships of BBB dysfunction and aging with microbiome alterations. However, we also acknowledge that other plausible neuroimmune mechanisms, such as altered gut-to-brain signals as mediated by the vagus nerve or the BSCFB, could also be contributing to CNS changes caused by gut microbiome dyshomeostasis [152,153].

### 5.4. Effects of Aging on Non-Endothelial Cells of the Neurovascular Unit

Endothelial cells of the BBB develop and maintain their specialized phenotype through interactions with other associated cell types in the CNS that include pericytes, astrocytes, neurons, and also other glial cell types such as microglia and oligodendrocytes [33]. Pericytes and astrocytes are the most extensively studied for their roles in promoting and maintaining BBB functions and may contribute to BBB dysfunction with aging. Detailed aspects of these changes are discussed below.

Pericyte loss/dysfunction: Numerous functions have been ascribed to brain pericytes, including contractility, pluripotent stem cell-like properties, phagocytosis, and induction and maintenance of the BBB [154,155]. Platelet-derived growth factor receptor beta (PDGFRβ) heterozygous mice, which show an age-dependent loss of brain pericytes, also have increased evidence of BBB disruption with aging which coincides with pericyte loss and precedes associated neuroinflammation and learning and memory impairment in this model [156]. In studies of wild-type mice and humans, pericyte loss has been reported with age, but not consistently [156,157,158,159]. However, it is more clear that pericyte damage can occur with age, perhaps through phagocytosis of increasing amounts of cell debris [159], which also occurs under inflammatory conditions [160]. Recent studies have reported an increase of soluble PDGFRβ in CSF, a proposed marker of pericyte damage, with aging, BBB disruption, and cognitive impairment in humans [91,161,162].

Astrocyte changes: Astrocyte endfeet ensheath brain capillaries, and contribute to BBB maturation and maintenance [65]. Phenotypic changes have been observed in astrocytes with aging, such as reduced vascular coverage, increased GFAP expression, enlarged size, and reduced aquaporin-4 (AQP4) expression [157,158,163]. Such changes indicate increased reactive astrogliosis, which is also a process that occurs in response to pro-inflammatory stimuli. Given the important role of AQP4 in facilitating paravascular clearance of brain solutes [164], AQP4 reductions on astrocytes with aging could contribute to the neurotoxic accumulation of solutes in the brain.

## 6. The BBB in Age-Associated Neurological Diseases

Aging increases the risk of developing disease, and many neurological conditions in which the BBB has been implicated are also associated with aging. This section discusses some of these diseases in context of age-associated BBB dysfunction that may predispose or exacerbate the molecular mechanisms of disease.

### 6.1. Alzheimer’s Disease

AD is the most common neurodegenerative disorder, and the greatest risk factor for AD is aging. There have been many recent reviews on the relations of BBB, inflammation, and AD that are beyond the scope of this review [57,165]. This section will focus on some recent conceptual advancements in the AD field that may relate inflammatory changes with aging and the BBB. 

Evolving concepts in AD: Under a new research framework proposed by the National Institute on Aging and Alzheimer’s Association, it was proposed that AD should be redefined by biological markers of disease, which include neurodegeneration and markers of deposition of two pathological proteins in the brain: amyloid beta and tau [166]. Whereas previous definitions of AD required a clinical diagnosis of dementia, it is now appreciated that pathological changes in AD precede onset of clinical symptoms by years, or even decades [167]. Although Aβ and tau are used to define AD as a unique neurodegenerative disease, it is now being considered that disease modifiers other than Aβ and tau may act in concert to regulate disease progression and manifestation of clinical symptoms. In previous sections, we have discussed aspects of BBB dysfunction that may be causal in AD, and here refer the reader to a recent detailed commentary on the importance of considering the neurovasculature as a possible driver of and therapeutic target for AD [104]. We also consider some additional timely findings that implicate interactions of the BBB, inflammation, and aging in AD.

ApoE isoform-dependent immunomodulatory activities: In humans, there are three major alleles of the apolipoprotein E (*APOE*) gene, which are *APOE2*, *APOE3*, and *APOE4*. *APOE4* is the strongest genetic risk factor for late-onset forms of Alzheimer’s disease, which may be due to a number of distinct functions of ApoE4 protein versus the more prevalent ApoE3 protein. ApoE4 may be contributing to AD in part via limiting Aβ clearance from the brain [168], and also through tau-dependent effects [169]. ApoE4 also has diverse functions in regulating the immune system that may be independent or synergistic with Aβ and tau-driven brain pathology [170]. For example, transgenic ApoE4 mice have BBB disruption through the loss of interaction of ApoE4 with LRP-1 in pericytes, which is preserved in mice expressing ApoE3 or ApoE2. As a result, matrix metalloproteinase 9 (MMP9) activation occurring in brain endothelial cells contributes to BBB disruption in the model [74]. This molecular route of BBB disruption in APOE4 carriers has also been reported in human AD [171]. 

Low-grade CNS infections and AD: Since the discovery of AD by Alois Alzheimer, there have been speculations and a few studies supporting that CNS infections could be causal in AD [172,173]. Although this concept has been largely overshadowed by the amyloid cascade hypotheses and is still controversial, emerging studies have supported that CNS bacterial and viral infections may contribute to or exacerbate AD. Early works have shown that herpes simplex virus-1 (HSV-1) DNA is present in brains of humans with and without AD [174,175], but it was also questioned whether HSV-1 infection was directly involved in AD [176]. Subsequently, it was shown that HSV-1 infection of cultured neurons and glia and mouse brain can increase the production of Aβ [177], and induce cytoskeletal abnormalities in neurons that include tau hyperphosphorylation [178]. Recent works have indicated that Aβ has antimicrobial properties against bacteria and viruses [179,180], and have substantiated the associations of herpes virus infections and AD or dementia [181,182,183,184,185]. These findings suggest that Aβ and/or tau may be protective responses to CNS infections that would be more likely to occur with age-associated immunosenescence and a dysfunctional BBB. It remains to be determined whether antimicrobial strategies such as antibiotics, antivirals, or vaccines could protect the infected against AD progression.

Preclinical animal models of AD: Mouse models of AD have been used extensively to define mechanisms of disease pathology and therapeutic efficacy. Most of these models are based on genetic mutations which cause Aβ plaque deposition, and so are really models of Aβ-driven brain injury [186]. However, other factors such as extraphysiological expression of transgenes and individual or combinations of mutations that are not observed in sporadic AD could further confound these models. Additionally, the AD-like sequalae (plaques, neuroinflammation, cognitive deficits, and neuronal/synaptic loss) in most transgenic mouse models of AD occur when the mice are considered to be young (3–6 mo.) or middle-aged (10–14 mo.), and so exclude the aging component of AD. Accelerated aging models, such as SAMP8 mice which have modest increases in brain Aβ, deficits in Aβ clearance, and impaired learning and memory by 12 months of age [187,188] are less widely used, but have utility in studying the synergy of aging and AD. Along these lines, BBB disruption variably occurs in mouse models of Aβ-driven brain pathology and is not apparent in SAMP8 mice [125,189,190,191,192], and recent works have also indicated that tauopathies may also drive BBB disruption in rodents [193,194]. BBB efflux systems have also been identified for truncated forms of tau [195]. Inclusion of an aging component in preclinical AD models may reveal important therapeutic considerations of treatment, or novel aspects of disease progression that may improve the chances of success in drug development.

### 6.2. Depression

Depression affects individuals of all ages, but poses unique considerations in the aging population. Although depression is less prevalent in older versus younger adults, it is notable that over half of depression diagnoses in the aged are in those who have not previously been afflicted [196]. Depression in the aged is also associated with cognitive dysfunction, dementia risk, and vascular dysfunction [196]. Notably, cardiovascular disease (CVD) and depression are inter-related in that major depressive disorder (MDD) prevalence is more prevalent in individuals with CVD, and MDD increases CVD morbidity and mortality [197]. Systemic inflammation may also be a factor that drives MDD, with studies showing associations of MDD and cytokines and acute phase proteins in blood [198,199]. The BBB has recently been implicated as a possible mediator of depressive behaviors in mice. Mice that were vulnerable to depressive-like behaviors following chronic social defeat stress were shown to have reductions in the tight junction protein claudin-5 and BBB leakiness in the nucleus accumbens (NAc), as well as increased leukocyte trafficking and IL-6 accumulation in this region. Knock-down of claudin-5 in the NAc recapitulated depressive-like behaviors [200]. Notably, aged mice have increased inflammatory responses to social defeat stress [201], suggesting that synergy of BBB dysfunction, glial cell priming, and increased peripheral cytokine responses could all contribute to depressive responses to stress in the aged. 

Another possible link between aging, the BBB, and depression is the microbiome. In humans with major depressive disorder, and in rodents subjected to a variety of stressors that can cause depressive-like behavior, composition of the gut microbiome is altered [202,203,204]. Humans with active MDD were shown to have an increase in Bacteroidetes and a reduction in Firmicutes, similar to age-associated microbiome changes that were discussed in Section 2.3. Recent work has also shown that the transplantation of microbiota from MDD patients into germ-free mice caused depressive-like behaviors, and altered metabolites of carbohydrates and amino acids [205]. Interestingly, the current data suggest that there is bidirectional regulation of the brain and gut microbiome in MDD. Future work is needed to determine how brain barriers may be contributing to gut-brain communication in MDD and other diseases.

### 6.3. Metabolic Syndrome

Metabolic syndrome is defined by a cluster of risk factors that increases risk of developing CVD, type II diabetes, stroke, and other co-morbid diseases [206,207]. These risk factors include insulin resistance, abdominal obesity, high serum triglycerides, high blood pressure, and hyperglycemia [208]. In the United States, metabolic syndrome is most prevalent in individuals aged 60 and older [209]. Age-associated factors such as low testosterone in males, and low levels of vitamin D may contribute to components of metabolic syndrome in the elderly, such as insulin resistance [209]. Testosterone depletion was recently linked to BBB dysfunction. Orchiectomized mice were shown to have increased BBB disruption, which was in part attributed to reduced expression of the tight junction proteins claudin-5 and zonula occludens-1. Castrated mice from this study also had evidence of reactive astrogliosis [210]. BBB deficits in castrated mice could be rescued by testosterone supplementation, but it was unclear whether the BBB effects occurred through direct actions of testosterone on the BBB, or indirect consequences of testosterone depletion. Notably, in mice, metabolic effects of orchidectomy are very minor or absent on a standard chow diet, but androgen deprivation can exacerbate adipose hypertrophy, glucose intolerance, insulin insensitivity, and systemic inflammation when fed a high-fat diet [211], suggesting that interactions between hormone changes with aging could synergize with diet and obesity to affect BBB disruption. 

Obesity is associated with increased systemic inflammation with aging and can exacerbate autoimmune diseases such as rheumatoid arthritis [212]. Obesity can also contribute to BBB dysfunction. Mice that are made to become obese through high-fat diet feeding have evidence of BBB disruption in the hippocampus, which is also associated with learning and memory deficits. BBB disruption in mice fed a high-fat also have reduced levels of tight junction proteins at the BBB and BCSFB [213]. Obesity is also associated with reduced transport of proteins across the BBB that act on the CNS to regulate feeding, such as leptin, insulin, and ghrelin. Whereas insulin and leptin signal satiety to the brain and stimulate anorexia, ghrelin is an orexigenic signal. However, levels of circulating leptin increase with obesity, whereas ghrelin levels decrease with obesity and aging [214]. In the case of ghrelin, age and obesity were also shown to have synergistic suppressive effects on transport across the BBB [215]. Transport of lipids such as palmitate and free fatty acids across the BBB is increased with obesity [213]. Triglycerides, which are elevated in obesity, are known inhibitors of leptin transport, and so may contribute to a feed-forward cycle of leptin deficiency in the brain that leads to hyperphagia and further increases in triglycerides [216]. Obesity induced by a genetic mutation in the leptin receptor has also been associated with increased neuroinflammation, which included monocyte trafficking across the BBB [217].

Type II diabetes mellitus (T2DM), a consequence of metabolic syndrome, is also associated with aging and BBB dysfunction. Studies in rodents and monkeys have shown that increased BBB disruption occurs in T2DM, and is associated with reduced levels of tight junction proteins [218,219,220]. T2DM is associated with an increased risk of AD [221], and recent work has demonstrated a mechanistic link that implicates BBB dysfunction in this process. In a mouse model of type II diabetes with hyperinsulinemia, it was shown that BBB transport of Aβ into the brain was increased, and transport of Aβ out of the brain was decreased when compared with non-diabetic controls. Antidiabetic drugs reduced Aβ influx and increased Aβ efflux in diabetic mice, and these changes appeared to be mediated through decreases of the Aβ influx transporter, receptor for advanced glycation endproducts (RAGE), and increases in the Aβ efflux transporter, LRP-1 [222]. The BBB dysfunction that occurs as a result of obesity alone, or in combination with T2DM may be further exacerbated by aging, or vice-versa [213,223,224]. 

## 7. Conclusions

Inflammatory changes with aging are important drivers of CNS dysfunction, and we have described mechanisms by which BBB dysfunction in healthy aging could predispose to neurological disease. Clearly, more work is necessary to further explore how aging and associated inflammatory changes could affect brain barrier functions in health, infection, and injury. Developing a better understanding of the interactions of aging with known pathogenic mechanisms of disease is important in the development of novel therapies for neurological disorders.
